# EBTM: An Enumeration-Based Thresholding Method for Degraded Circular Dot Segmentation

**DOI:** 10.3390/s25072158

**Published:** 2025-03-28

**Authors:** Baoquan Shi, Qian He, Xianmin Chen, Wendong Zhang, Lin Yang

**Affiliations:** 1 School of Mechano-Electronic Engineering, Xidian University, Xi’an 710071, China; 2 National Key Laboratory of Strength and Structural Integrity, Xi’an 710065, China; heqianrun@163.com (Q.H.); vitochan@163.com (X.C.); dongzi.666@163.com (W.Z.); 3 Xi’an Baochuang Suwei Intelligent Research Co., Ltd., Xi’an 712000, China; 4 AVIC Aircraft Strength Research Institute of China, Xi’an 710065, China; 5 National Wind Energy Testing & Certification (Tianjin) Co., Ltd., Tianjin 300462, China; yanglin@nwetc.com.cn

**Keywords:** threshold, degraded circular dot, enumeration

## Abstract

Circular dots are widely used in various measurement applications due to their inherent symmetry, ease of detection, and scalability. However, when degraded by factors such as specular highlights, low contrast, strong noise, or friction damage, accurately extracting them from the background becomes a significant challenge. To address this issue, an enumeration-based thresholding method (EBTM) is proposed for degraded circular dot segmentation. Firstly, a series of candidate outputs are generated using an enumeration-based thresholding scheme. Next, an assessment criterion is developed to evaluate these candidate outputs. Finally, the optimal segments are selected from each candidate output and combined to produce a reasonable thresholding result. Unlike traditional methods, the novel approach does not focus on selecting the optimal threshold values, but instead aims to choose the best segments to produce the desired output. Owing to the enumeration-based thresholding mechanism, the novel approach demonstrates greater robustness in handling the challenges in degraded circular dot images. Extensive comparative studies demonstrate the superiority of the novel approach.

## 1. Introduction

Circular dots have the characteristics of inherent symmetry, ease of detection, and scalability. The well-defined geometry of circular dots makes them ideal for use as measurement targets, which is essential for a wide range of applications [1,2,3,4,5,6,7,8]. In photogrammetry, circular dots function as fiducial markers, facilitating the alignment and calibration of images captured from different perspectives [1]. Similarly, in motion capture systems, circular reflective markers are attached to subjects to track their movements in three-dimensional space with high accuracy [2]. Circular dots can also be projected onto surfaces, where their deformation caused by surface topography is analyzed to measure surface roughness or reconstruct the 3D shape of an object [5]. Additionally, in materials science and engineering, circular dots are applied to materials to measure deformation or strain fields under stress [6,7,8], providing critical insights into material behavior under load. In summary, the geometric properties of circular dots make them a versatile and reliable tool for accurate data acquisition across a wide range of fields.

**Figure 1:**
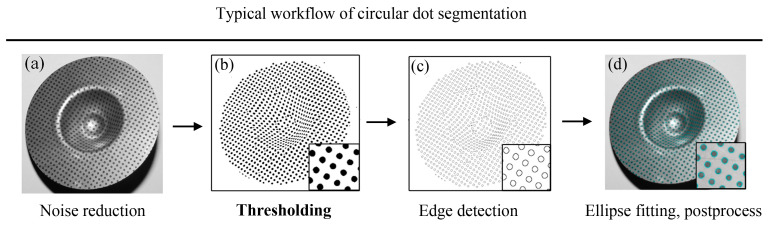
Typical workflow of circular dot segmentation [8]. (**a**) Noise reduction applied to the original dot image. (**b**) Thresholding result. (**c**) Detected edges of the circular dots. (**d**) Ellipse fitting and postprocessing.

Segmentation of circular dots from the background is a fundamental operation in image processing, particularly for tasks such as edge extraction (both at the pixel and subpixel levels) and center detection. As shown in Figure 1, a typical workflow includes the following steps [8]: (1) Noise reduction: apply a Gaussian blur or median filter to reduce noise while preserving the edges of the circular dots. (2) Thresholding: convert the image to a binary format using techniques such as global thresholding or locally adaptive thresholding to extract the circular dots from the background. (3) Pixel-level and subpixel edge detection: use edge detection algorithms to extract the edges of the circular dots. Refine the edge locations to subpixel accuracy using techniques such as Gaussian fitting of edge profiles or interpolation-based methods (e.g., bilinear or bicubic interpolation). (4) Ellipse fitting: fit an ellipse to the edge points of each circular dot using least squares or RANSAC for robust fitting. The center of the ellipse is then identified as the detected center. (5) Postprocessing: remove false detections or outliers using statistical methods (e.g., based on size, intensity, or position). If the circular dots are well imaged, they can be accurately extracted from the background using the methods described above. However, for degraded circular dots—such as those affected by strong noise, low contrast, uneven illumination, or friction damage—segmentation becomes significantly more challenging [7]. These degradations can lead to inaccurate thresholding, which in turn affects the reliability of edge extraction and center detection.

This paper focuses on the thresholding/binarization step, which is critical for accurately separating circular dots, particularly in cases where the circular dots are degraded. By addressing the challenges associated with degraded circular dot images and improving the thresholding process, the overall accuracy and reliability of subsequent operations—such as edge extraction (both pixel-level and subpixel-level) and center detection—can be significantly enhanced.

### 1.1. Related Works

Thresholding, a fundamental technique in image processing, serves as the most straightforward approach for image segmentation by distinguishing object pixels from background pixels [9,10]. Based on the number of pixel groups generated, thresholding methods are systematically categorized into two primary types: bilevel thresholding and multilevel thresholding [11]. Bilevel thresholding, commonly referred to as binarization, transforms grayscale images into binary images by dividing pixels into two distinct groups: foreground and background. This category can be further subdivided into three methodologies: global thresholding, local thresholding, and learning-based thresholding.

Global thresholding methods segment an image into foreground and background using a single threshold value [12]. Over the years, numerous techniques have been developed to determine an optimal global threshold, including histogram-based methods [13], entropy-based methods [14], and clustering-based methods [15], among others. Comprehensive reviews and comparative analyses of these methods are available in reference [16]. While global thresholding is straightforward and parameter-free, its reliance on a single threshold makes it susceptible to variations in noise, contrast, illumination, and shadows. Despite these limitations, its simplicity and robustness have made it a popular choice in applications such as document binarization, computer vision, and pattern recognition. Notably, Otsu’s method [13], one of the most effective global thresholding techniques, along with its variants [17], is frequently employed as a preprocessing step in computer vision and image understanding due to its reliable performance in real-world image segmentation tasks [18].

Local thresholding, also known as dynamic or adaptive thresholding [19], determines a threshold value for each pixel based on local statistical properties such as range, variance, or contrast. Niblack [20] pioneered an algorithm that calculates thresholds using local mean and standard deviation. Sauvola and Pietikainen [21] introduced a hybrid approach combining a soft decision method for background and pictorial regions with a specialized text binarization method for textual and line-drawing areas. Gatos et al. [22] developed a comprehensive binarization framework involving preprocessing, rough foreground estimation, background surface calculation, and thresholding. Su et al. [23] proposed a novel local thresholding technique based on local image maxima and minima. Jia et al. [17] employed structural symmetric pixels to compute local thresholds within neighborhoods, followed by pixel classification using a voting strategy. Howe [24] introduced a method leveraging the Laplacian energy of image intensity, incorporating a stability heuristic to optimize parameter selection for individual images. These local thresholding methods exhibit robustness against nonuniform illumination, shadows, low contrast, and noise, making them particularly effective in document image binarization and optical character recognition (OCR) [25].

Learning-based thresholding methods have emerged as a powerful approach for extracting object pixels from the background in an image [26]. These methods are based on convolutional neural networks (CNNs) or other deep learning frameworks [27,28]. For instance, He and Schomaker [29] developed an iterative deep learning framework for document enhancement and binarization. Castellanos et al. [30] proposed a method that combines neural networks and domain adaptation techniques to perform unsupervised document binarization. However, this approach may not be effective when the source and target domains are very similar. To address these challenges, Suh et al. [31] proposed a two-stage color document image enhancement and binarization method using generative adversarial neural networks. Similarly, Yang and Xu [32] proposed an end-to-end binarization model through vision transformer, which autonomously optimizes its parameterized configuration of the entire learning pipeline without incurring the intensity-to-binary value conversion phase, resulting in improved binarization quality. Despite their effectiveness, learning-based methods require a large amount of data and computation resources to train the network [33]. Nonetheless, these methods have the advantage of performing thresholding without the need to calculate any threshold values.

Multilevel thresholding methods employ two or more threshold values to partition images into multiple classes. Yen et al. [34] proposed a novel criterion for multilevel thresholding that considers the discrepancy between segmented and original images, along with the bit representation requirements of the segmented image. Arora et al. [35] utilized image mean and variance to determine optimal thresholds for multilevel segmentation. Pare et al. [36] conducted an extensive review of approximately 157 significant studies on multilevel thresholding-based image segmentation, analyzing various objective functions. These methods provide richer information through segmented images, making them highly valuable in image segmentation frameworks. However, as the number of segmentation levels increases, so does the computational complexity, presenting a trade-off between detail and efficiency.

### 1.2. Our Work

**Figure 2:**
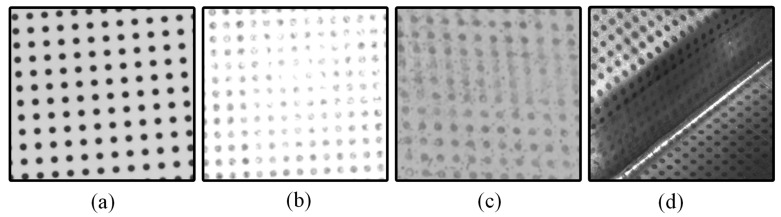
Circular dots: (**a**) clearly imaged with high contrast; (**b**) degraded by specular highlights; (**c**) degraded by low contrast and strong noises; (**d**) degraded by friction damage introduced during stamping or forming process.

In general, when circular dots are clearly imaged with high contrast, as shown in Figure 2a, it is possible to accurately separate the circular dots from the background using either a single threshold or a combination of multiple thresholds. However, in real circular dot images, as shown in Figure 2b–d, several factors can complicate this process. These include specular highlights caused by the strong reflectivity of the object’s surface, low contrast, strong noise introduced during data acquisition, and friction damage resulting from mechanical stress (such as stamping or forming processes). These issues can significantly degrade the quality of the circular dots [7], making it a considerable challenge to accurately extract them from the background.

To overcome these challenges, this paper introduces an enumeration-based thresholding method (EBTM) designed specifically for degraded circular dot segmentation. The approach consists of several key steps to achieve accurate segmentation. (1) Candidate generation: instead of determining an optimal threshold for each pixel individually, the novel approach presents an enumeration-based thresholding scheme that systematically generates a series of candidate outputs. (2) Segments assessment: a dedicated assessment criterion is developed to evaluate the quality of each segment in the candidate output. This metric considers factors such as ellipse-fitting errors and the preservation of circular dot shape and size, enabling the identification of the segments that best meet the desired quality standards. (3) Optimal segments screening and combination: the candidate outputs are screened to select the optimal segments, which are then combined to produce the final thresholding result. This process ensures that the final output incorporates the best possible segments obtained through enumeration.

The novel approach functions as a bilevel thresholding technique by effectively separating the circular dots (foreground) from the background. Its advantages, when compared to other bilevel thresholding methods [12,13,14,15,16,17,18,19,20,21,22,23,24,25], can be highlighted from several perspectives: (1) Robustness to illumination variations. Unlike global thresholding methods [12,13,14,15,16,17,18], which apply a single threshold to the entire image, the proposed method operates locally on each pixel. This localized approach enhances its robustness against nonuniform illumination and shadows. (2) Resilience to low contrast, specular highlights, and strong noise. Traditional local thresholding methods [20,21,22,23,24,25] often rely on local statistics such as range, variance, or contrast, which can be adversely affected by low contrast, specular highlights, and strong noise. In contrast, by enumerating possible segments through an iterative process, the proposed approach is less sensitive to these issues. (3) Simplicity and computational efficiency. Compared to learning-based thresholding methods [26,27,28,29,30,31,32,33]—which typically involve complex algorithms and high computational demands—the proposed approach is straightforward to implement and requires significantly lower computational resources.

A comparative study using real circular dot images validates the good performance of the novel approach. The contributions of this research are summarized as follows: (1) Introduction of a novel methodology. A new enumeration-based thresholding method is presented, tailored specifically for circular dot segmentation. (2) Improved segmentation quality. By employing an enumeration thresholding scheme and a robust segmentation screening criterion, the method achieves more accurate and reliable segmentation results. (3) Enhanced robustness. The approach effectively handles various challenges—including nonuniform illumination, shadows, low contrast, specular highlights, noise, and friction damage. (4) Demonstrated superiority. Comparative studies with real circular dot images show that the proposed approach significantly increases the number of accurately separated circular dots, outperforming existing methods.

In summary, this paper offers a novel, efficient, and robust enumeration-based thresholding approach that addresses the unique challenges of degraded circular dots segmentation, providing superior performance in various challenging imaging conditions.

## 2. Enumeration-Based Thresholding Method (EBTM)

In this section, we begin with a detailed explanation of the enumeration-based thresholding principle. Next, we introduce the segment assessment criterion along with the optimal segment screening and combination rule, highlighting their key aspects. Finally, we outline the implementation details of the proposed novel approach.

### 2.1. Candidate Generation

The fundamental principle of local thresholding is to determine an appropriate threshold value T(x,y) for each pixel (x,y) in a grayscale image, thereby classifying the pixel as either belonging to the background or the foreground. This can be expressed as follows:

(1) $$\Psi (x,y)=\left\{\begin{array}{cc} 0 & if\;I(x,y)<T(x,y)\\ 255 & if\;I(x,y)\geq T(x,y) \end{array}\right.,$$

where *x* and *y* represent the spatial coordinates of the image, I(x,y) is the intensity or gray value at pixel (x,y), and Ψ(x,y) is the thresholding output.

The formation of a visual image, represented by a gray level function I(x,y), can be modeled as the product of an illumination component i(x,y) and a reflectance component r(x,y) [37]:

(2) I(x,y)=i(x,y)×r(x,y).

From Equation (2), it follows that two primary types of information are conveyed through an image. The first type, carried by i(x,y), pertains primarily to the lighting of the scene. The second type, represented by r(x,y), is concerned entirely with the characteristics of the objects within the scene. Although both components are presented together, they convey distinct messages, each reflecting different aspects of the scene. Unfortunately, the functional behavior of i(x,y) and r(x,y) is not explicitly known. Therefore, the threshold function T(x,y) is generally expressed as a function of I(x,y) [38]:

(3) T(x,y)=T(I(x,y)).

Since i(x,y) and r(x,y) are two independent components in terms of the nature of the information they convey, their contributions to the threshold function T(x,y) can be evaluated independently as T(i(x,y)) and T(r(x,y)), i.e., T(I(x,y))=T(i(x,y))+T(r(x,y)). By denoting T(i(x,y)) and T(r(x,y)) as $T_{i}(x,y)$ and $T_{r}(x,y)$, respectively, Equation (3) can be expressed as follows:

(4) $$T(x,y)=T_{i}(x,y)+T_{r}(x,y).$$

In general, the illumination component i(x,y) varies slowly over space, while the reflectance component r(x,y) primarily contains spatially high-frequency details [34]. Consequently, the subfunction $T_{i}(x,y)$ should closely track small changes in i(x,y), whereas the subfunction $T_{r}(x,y)$ should closely track large changes in r(x,y).

The function forms of $T_{i}(x,y)$ and $T_{r}(x,y)$ are unknow, but they can be approximated using local statistics. In a local region, the local mean is a stable local statistic of pixel intensity that changes gradually from one region to another. It can be used to approximate the subfunction $T_{i}(x,y)$:

(5) $$T_{i}(x,y)\approx m(x,y)=\frac{1}{w^{2}}\sum_{p=x-w/2}^{x+w/2}\sum_{q=y-w/2}^{y+w/2}I(p,q),$$

where w represents the size of region or window centered around the pixel (x,y).

The local standard deviation is another local statistic that measures the variation in pixel intensity within a region or window. As such, it can be used to approximate the subfunction $T_{r}(x,y)$. In fact, in some locally adaptive thresholding methods, the threshold function T(x,y) is expressed as a function of the local mean m(x,y) and local standard deviation. In Niblack’s method [20], T(x,y)=m(x,y)+κ×δ(x,y), where κ is a bias and δ(x,y) is the local standard deviation. Here, $T_{r}(x,y)$ can be approximated as $T_{r}(x,y)\approx \kappa \times \delta (x,y)$. In Sauvola’s method [21], T(x,y)=m(x,y)+k×m(x,y)×(δ(x,y)/R−1), where *k* is a user-defined parameter and *R* denotes the dynamic range of the standard deviation (usually set to 128 for grayscale images). In this case, $T_{r}(x,y)$ can be approximated as $T_{r}(x,y)\approx k\times m(x,y)\times (\delta (x,y)/R-1)$. These locally adaptive thresholding methods generally yield better segmentation results than global thresholding methods in most applications [39], as they incorporate both the local mean and local standard deviation into the thresholding function T(x,y). It can be inferred that if the two subfunctions $T_{i}(x,y)$ and $T_{r}(x,y)$, particularly the latter, can more accurately describe the variations caused by the illumination component i(x,y) and the reflectance component r(x,y), the thresholding output will be improved. However, this paper does not focus on developing a new functional form for $T_{r}(x,y)$. Instead, it adopts an enumeration strategy to list possible values of $T_{r}(x,y)$, generates a series of candidate thresholding outputs, and ultimately selects the optimal segments in the candidate outputs.

The value of r(x,y) is bounded within [0.005, 1]. Therefore, the value of the subfunction $T_{r}(x,y)$ should also be constrained, i.e., $T_{r,\min}(x,y)\leq T_{r}(x,y)\leq T_{r,\max}(x,y)$, where $T_{r,\min}(x,y)$ and $T_{r,\max}(x,y)$ represent the minimum and maximum values of $T_{r}(x,y)$. Additionally, within a local region or window, the thresholding value of T(x,y) should be bounded within $\left[I_{\min}(x,y),I_{\max}(x,y)\right]$, where $I_{\min}(x,y)$ and $I_{\max}(x,y)$ are the local minimum and maximum pixel intensities, respectively. Thus, the following equation can be established:

(6) $$\left\{\begin{array}{c} T_{r,\min}(x,y)=I_{\min}(x,y)-T_{i}(x,y)\approx I_{\min}(x,y)-m(x,y)\\ T_{r,\max}(x,y)=I_{\max}(x,y)-T_{i}(x,y)\approx I_{\max}(x,y)-m(x,y) \end{array}\right..$$

That is, $T_{r}(x,y)$ is bounded within $\left[I_{\min}(x,y)-m(x,y),I_{\max}(x,y)-m(x,y)\right]$. Moreover, since the pixel intensity values of I(x,y) are integers, the value of $T_{r}(x,y)$ can take only integer values within the lower and upper bounds of this range. Assuming the integer values of $T_{r}(x,y)$ form a new domain set $H=\left\{h_{j}\right\}$, 1≤j≤n, where *n* is the size of the domain set *H*, $h_{j}$ is an integer threshold value, and $T_{r,\min}(x,y)\leq h_{j}\leq T_{r,\max}(x,y)$. Consequently, Equation (4) can be expressed as follows:

(7) $$T(x,y)=T_{i}(x,y)+T_{r}(x,y)\approx m(x,y)+h_{j}.$$

And Equation (1) can be expressed as follows:

(8) $$\Psi_{j}(x,y)=\left\{\begin{array}{cc} 0 & if\;I(x,y)<m(x,y)+h_{j}\\ 255 & if\;I(x,y)\geq m(x,y)+h_{j} \end{array}\right..$$

**Figure 3:**
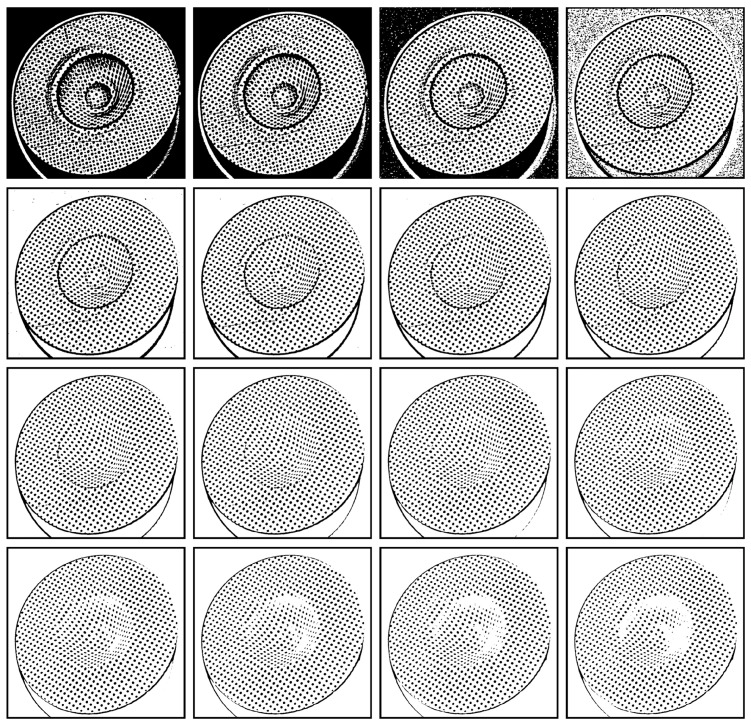
Candidate thresholding outputs of the circular dot image in Figure 1a under different values of *h_j_*. From top left to bottom right, the value of *h_j_* is set to −5, −3, −1, 1, 3, 5, 7, 9, 11, 13, 15, 17, 19, 21, 23, and 25, respectively.

A set of candidate outputs $\Psi_{j}(x,y)$ can be generated based on Formula (8), and the optimal thresholding output $\Psi^{opt}(x,y)$ is guaranteed to be included within this set, i.e., $\Psi^{opt}(x,y)\in \{\Psi_{1}(x,y),\Psi_{2}(x,y),\ldots ,\Psi_{n}(x,y)\}$. Figure 3 gives an example where the local mean *m*(*x*, *y*) was computed within a 25 × 25 window centered around each pixel. The value of *h_j_* was iteratively enumerated from −5 to 25, resulting in approximately 31 candidate thresholding outputs, from $\Psi_{1}(x,y)$ to $\Psi_{31}(x,y)$) (only 16 candidates were listed in the figure). Notably, nearly all of the circular dots were correctly separated. However, some were accurately separated in candidate outputs with smaller values of *h_j_*, while others were accurately separated in candidate outputs with larger values of *h_j_*.

### 2.2. Segment Assessment

To identify the optimal segments from the candidate outputs, it is essential to establish a criterion for evaluating the quality of the segments. Given the assumption that the segments are circular or elliptical in shape, an ellipse-fitting algorithm can be utilized to indirectly assess the quality of the segments [7]. The process consists of the following steps: (1) Clustering. The segments are grouped such that pixels belonging to the same circular dot are clustered together. The depth-first search (DFS) algorithm is used for this purpose. The algorithm starts from an unvisited pixel and recursively visits all adjacent pixels until all regions connected to the current pixel are labeled. (2) Edge Extraction. The edge pixels of each segment are identified using the Moore neighbor contour tracing algorithm. This algorithm traces the contours of a segment by analyzing the connectivity of neighboring pixels. A pixel is classified as an edge pixel if at least one of its eight neighboring pixels is empty (i.e., not part of the segment). (3) Ellipse Fitting. An ellipse-fitting algorithm is applied to the extracted edge pixels. This algorithm fits an ellipse to the edge pixels based on their spatial distribution. The fitting error, which quantifies the deviation between the fitted ellipse and the actual edge pixels, serves as a metric to evaluate the quality of the segments in candidate outputs. By leveraging this approach, the quality of the segments can be indirectly estimated through the fitting error derived from the ellipse-fitting algorithm. This enables the extraction of the optimal segments from the candidate outputs.

Considering computational efficiency, the direct least square ellipse-fitting algorithm [40] is employed in this approach. The general form of an ellipse can be expressed as follows:

(9) $$f(\beta )=Ax^{2}+Bxy+Cy^{2}+Dx+Ey+F=0,$$

where β=(A,B,C,D,E,F) represents the ellipse parameters. Denoting the detected edge pixels of a segment as $p_{k}=(x_{k},y_{k})$, k=1,2,…,m, m is the number of extracted edge pixels, the corresponding least squares optimization function can be formulated as follows [40]:

(10) $$\left\{\begin{array}{c} \min\sum_{k=1}^{m}f{\left(\beta \right)}^{2}\;\;\;\;\;\\ s.t.\;B^{2}-4AC=1\; \end{array}\right.,$$

By solving Equation (10), the ellipse parameters β can be determined. Subsequently, the fitting error at each edge pixel $p_{k}$ is calculated as follows:

(11) $$e_{k}=\;Ax_{k}^{2}+Bx_{k}y_{k}+Cy_{k}^{2}+Dx_{k}+Ey_{k}+F.$$

The fitting error $e_{k}$ represents the squared distance between the *k*-th edge pixel and the fitted ellipse. By computing the fitting errors for all edge pixels, the overall quality of the segment can be assessed. A smaller fitting error indicates a better fit between the ellipse and the extracted edge pixels, implying a more accurate segmentation of the corresponding circular dot. To comprehensively evaluate the quality, both the average fitting error $e_{mean}$ and the maximum fitting error $e_{\max}$ are calculated for each segment:

(12) $$\left\{\begin{array}{c} e_{mean}=\sum_{k=1}^{m}e_{k}/m\;\;\;\;\;\;\\ e_{\max}=maximum(e_{k})\; \end{array}\right..$$

Additionally, as shown in Figure 3, an increase in the value of $h_{j}$ may lead to a reduction in the areas of some segments. To assess the extent of this area shrinkage, a shrinkage ratio α is calculated for each segment:

(13) $$\alpha =\left|4\times N-\pi d^{2}\right|/\left(\pi d^{2}\right),$$

where *N* denotes the size of the segment (equivalent to the area of the segment), and *d* is the diameter of the corresponding circular dot in the original image. The shrinkage ratio is computed by dividing the area of the segments by the area of the corresponding circular dot. Typically, the shrinkage ratio α is larger for segments with significant shrinkage and smaller for those with minimal shrinkage. For a more comprehensive evaluation, the shrinkage ratio α is combined with the other two factors to derive a synthetic error:

(14) $$e_{total}=0.2\times e_{mean}+0.2\times e_{\max}+0.6\times \alpha .$$

In conclusion, three factors, $e_{mean}$, $e_{\max}$, and $e_{total}$, are calculated to assess the quality of segments in the candidate outputs. For homogeneous segments derived from different candidate outputs, smaller values of these factors indicate better quality of the segments. Using this criterion, the optimal segments can be selected to generate the desired thresholding output.

### 2.3. Optimal Segment Screening and Combination

To obtain the final thresholding output, the segments in the candidate outputs are evaluated based on the criterion described in Formula (14). The optimal segments, denoted as $\Psi^{opt}(x,y)$, are then selected. These optimal segments are subsequently combined to generate the desired thresholding result:

(15) $$\Psi (x,y)=\cup \Psi^{opt}(x,y).$$

**Figure 4:**
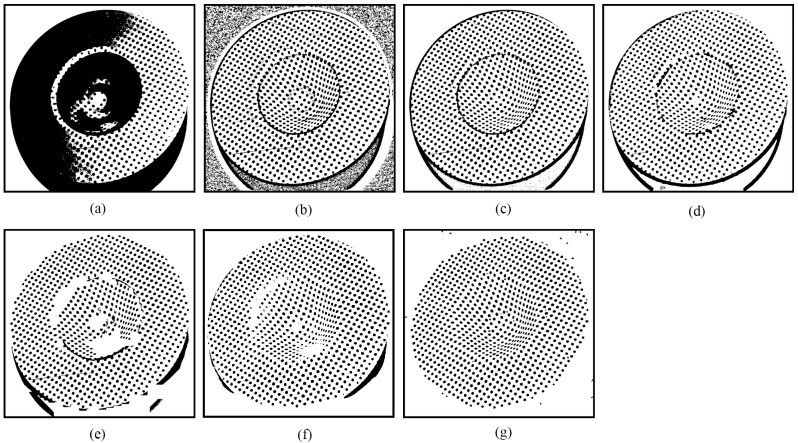
Thresholding output of the circular dot image shown in Figure 1a: (**a**) Otsu [13]; (**b**) Niblack [20]; (**c**) Sauvola [21]; (**d**) Su [23]; (**e**) Jia [24]; (**f**) U2-Net [27]; and (**g**) EBTM.

Figure 4g shows the final thresholding output of the circular dot image originally shown in Figure 1a. This output consists of the optimal segments selected from the candidate outputs presented in Figure 3. Notably, the novel approach achieves superior thresholding performance compared to both the global thresholding method of Otsu [13] and the locally adaptive thresholding methods of Niblack [20], Sauvola [21], Su [23], Jia [24], and U2-Net [27], highlighting the effectiveness of the novel thresholding method.

### 2.4. Implementation Detail of the Novel Approach

The novel approach processes grayscale images, and any color image must first be converted to grayscale before thresholding. Additionally, to reduce image noise, a median filter with a 3 × 3 or 5 × 5 window can be applied as a preprocessing step.

The window size *w* should be large enough to ensure that the calculated local mean *m*(*x*, *y*) varies smoothly within local regions. However, a larger window size will inevitably increase the computational burden. To strike a balance, the window size *w* can be calculated as follows:

(16) w=d+c,

where *d* represents the diameter of the circular dots in the image, and *c* is a constant mainly used to ensure the window size *w* is large enough to cover the circular dots. In our implementation, if *d* is an even number, the constant *c* is set to 7; otherwise, it is set to 8 so that window size *w* is always an odd number, and thus, its center can align with the pixel being processed, maintaining a symmetric neighborhood. The diameter *d* should be manually specified. According to experiments, the influence of *d* on the thresholding results is minimal; thereby, it is randomly set as the diameter of a larger circular dot.

The value of *h_j_* has a significant impact on the candidate thresholding output. A small value of *h_j_* would cause some segments to stick together, as shown in the second row of Figure 3. Furthermore, a smaller value of *h_j_* would introduce noise in the background (first row of Figure 3), which may ultimately be included in the thresholding output, as shown in Figure 4g. On the other hand, a larger value of *h_j_* would shrink the areas of some segments, although it could separate other segments that were initially sticking together, as shown in the fourth row of Figure 3. Moreover, the value of *h_j_* should be positive since negative values would lead to false segmentation of the background, as depicted in the first row of Figure 3. Here, an adaptive procedure is developed to determine the value of *h_j_* as follows:

Step 1. Determine the lower bound of *h_j_*. As mentioned above, the value of *h_j_* should be positive, thereby the lower bound of *h_j_* is set to 1 (i.e., *h*_1_ = 1).

Step 2. Determine the initial upper bound of *h_j_*. According to the lower bound of *h_j_*, the candidate thresholding output $\Psi_{1}(x,y)$ can be generated according to Formula (8), and the average local mean $\tilde{m}(x,y)$ as well as the maximum pixel intensity $I_{\max}(x,y)$ can be obtained. Set the optimal threshold output to $\Psi^{opt}(x,y)=\Psi_{1}(x,y)$. According to the inequality $h_{j}\leq T_{r,\max}(x,y)$, the upper bound of *h_j_* can be initialized as $I_{\max}(x,y)-\tilde{m}(x,y)$.

Step 3. Enumeration and update. Generate the *j*th candidate threshold output $\Psi_{j}(x,y)$ by enumerating the value of *h_j_* within its lower and upper bounds, and update $\Psi^{opt}(x,y)$. In each enumeration, *h_j_* is set to $h_{j-1}+2$, and the quality of each segment in $\Psi_{j}(x,y)$ is checked. If the quality of a new segment is higher than that of its corresponding segment in $\Psi^{opt}(x,y)$, replace the corresponding segment in $\Psi^{opt}(x,y)$ with the new segment. Moreover, if a corresponding segment cannot be found for the new segment, simply insert the new segment into $\Psi^{opt}(x,y)$.

Step 4. Judgment. If $\Psi^{opt}(x,y)$ is updated, repeat step 3. Otherwise, stop the enumeration.

Although the upper bound of *h_j_* is initialized as $I_{\max}(x,y)-\tilde{m}(x,y)$ in step 2, its true value may be smaller if all optimal segments are found earlier, causing the enumeration to stop. That is, the actual upper bound of *h_j_* is determined dynamically. The worst case occurs when the enumeration stops at the initial upper bound. Extensive experiments indicate that the optimal threshold output can be obtained after only several enumerations or, in some cases, around a dozen.

## 3. Results and Discussion

To better evaluate the performance of the novel approach, a comparative study was conducted using the global thresholding method of Otsu [13], the locally adaptive thresholding methods of Niblack [20], Sauvola [21], Su [23], and Jia [24], as well as the learning-based thresholding network U2-Net [27]. The methods of Otsu [13], Niblack [20], Sauvola [21], and Su [23] were implemented in C++. For the thresholding method proposed by Jia [24], the executable program [41] provided by the authors were used. For the learning-based thresholding network U2-Net [27], the source code [42] provided by the authors were used.

The parameter of the window size w for Niblack [20], Sauvola [21], and Su [23] was set to 30, while the parameter k for Sauvola [21] was set to 0.1. All other parameters were configured to the default values recommended by the authors and/or developers. For U2-Net [27], the database DIBCO [43] for document image binarization, and the dataset CryoNuseg [44] for nuclei segmentation were employed to train the network. The test data comprised real circular dot images obtained from practical sheet metal forming strain measurement applications.

Compared to existing methods, the novel approach demonstrates greater robustness against nonuniform illumination and specular highlights. As shown in Figure 4, most of the segmented circular dots within the areas affected by specular highlights are either grouped together, as shown in Figure 4a–d, or discarded, as shown in Figure 4e. Although the U2-Net [27] correctly separated some circular dots at the regions with nonuniform illumination and specular highlights, the others are still missed, as shown in Figure 4f. Encouragingly, owing to the enumeration thresholding strategy and the optimal segment selection mechanism, nearly all circular dots are successfully separated using the novel approach, as shown in Figure 4g.

**Figure 5:**
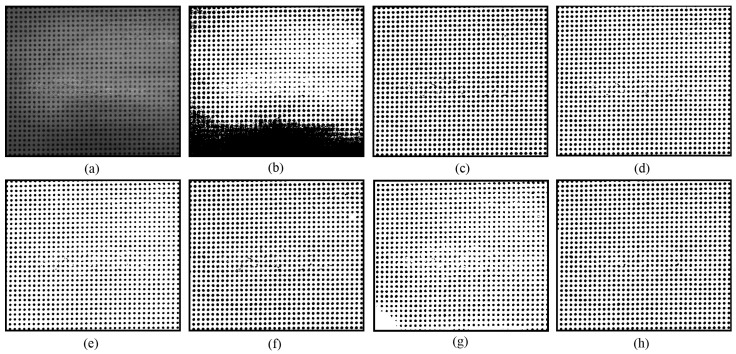
Thresholding of a circular dot image with some dots damaged by slight specular highlights: (**a**) original; (**b**) Otsu [13]; (**c**) Niblack [20]; (**d**) Sauvola [21]; (**e**) Su [23]; (**f**) Jia [24]; (**g**) U2-Net [27]; and (**h**) EBTM.

Figure 5 presents another example where some circular dots are affected by mild specular highlights. It can be seen that Otsu’s method [13] produces poor thresholding results, as shown in Figure 5a. The locally adaptive thresholding methods of Niblack [20], Sauvola [21], Su [23], Jia [24], and U2-Net [27] generate better thresholding outputs than Otsu’s method, but the segmented circular dots within the specular highlight areas are still plagued by considerable noise (Figure 5b–g). In contrast, the novel approach achieves the best thresholding output, with circular dots effectively separated from either specular highlight areas or uniform illumination areas, as shown in Figure 5h.

**Figure 6:**
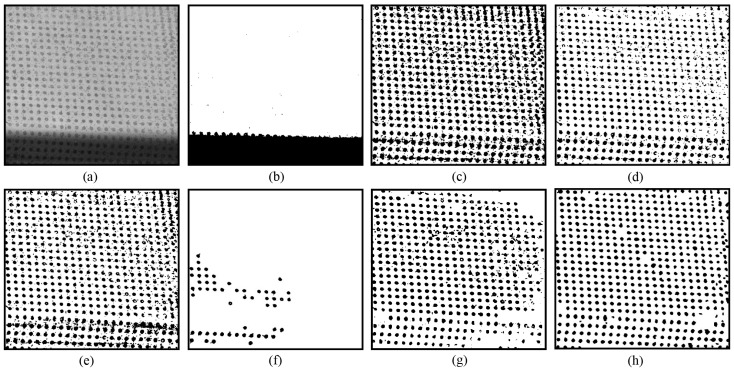
Thresholding of a circular dot image with low contrast and strong noise: (**a**) original; (**b**) Otsu [13]; (**c**) Niblack [20]; (**d**) Sauvola [21]; (**e**) Su [23]; (**f**) Jia [24]; (**g**) U2-Net [27]; and (**h**) EBTM.

The novel approach also demonstrates robustness against low contrast and strong noise. Figure 6 provides an example where some circular dots exhibit low contrast, while others are blurred by intense noise. It can be seen that Otsu’s method [13] fails to separate any circular dots, as shown in Figure 6b. This may be due to the histogram being influenced by the shadow at the bottom of the image. For Jia’s method [24], a better thresholding result was obtained with a parameter setting of Rthre = 0.01, but only a few circular dots were separated correctly, as shown in Figure 6f. The outputs from the methods of Niblack [20], Sauvola [21], Su [23], and U2-Net [27] contain substantial noise, as shown in Figure 6c–e,g. In contrast, the novel approach produces promising thresholding results, with most of the circular dots correctly separated, as shown in Figure 6h.

**Figure 7:**
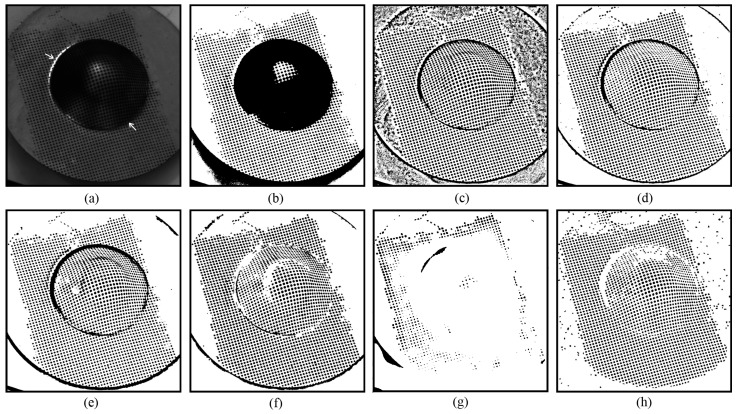
Thresholding of a circular dot image with shadow: (**a**) original; (**b**) Otsu [13]; (**c**) Niblack [20]; (**d**) Sauvola [21]; (**e**) Su [23]; (**f**) Jia [24]; (**g**) U2-Net [27]; and (**h**) EBTM.

The novel method is also robust to shadows and friction damages resulting from mechanical stress (such as the stamping/forming process). Figure 7 presents an example where some circular dots were damaged by the punch load during stamping process. The damaged circular dots present a particular challenge for thresholding methods due to their low contrast, incomplete shapes, and placement in regions with shadows and specular highlights (as indicated by the arrows in Figure 7a). It can be seen that U2-Net [27] performs poorly. A likely reason is that the circular dots in Figure 7 are smaller than those in the other tested images, suggesting that U2-Net [27] lacks robustness when segmenting small objects. Additionally, Otsu’s method [13] performs poorly in the shadow regions. The methods of Niblack [20], Sauvola [21], Su [23], and Jia [24] yield better results in the shadow regions, but the damaged circular dots are rarely correctly separated, often being either grouped together or discarded (Figure 7c–g). In contrast, the novel approach produces the best results: most of the circular dots in the shadow regions are correctly separated, and a significant number of damaged circular dots are also accurately extracted, as shown in Figure 7h. It can also be observed in Figure 7h that some noise remains in the background, introduced by a smaller value of *h_j_* during the enumeration procedure (see Section 2.4). Increasing the lower bound of *h_j_* would help reduce this noise.

**Figure 8:**
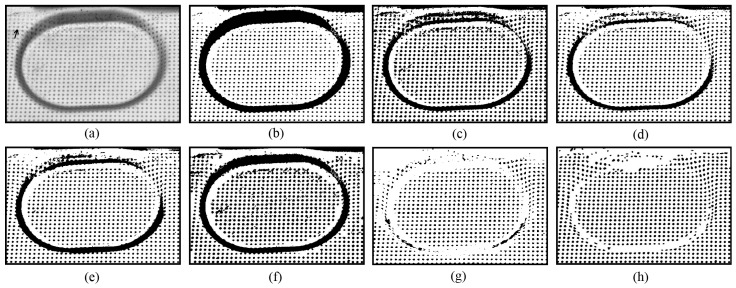
Thresholding of a circular dot image with some dots damaged by friction introduced during the stamping/forming process: (**a**) original; (**b**) Otsu [13]; (**c**) Niblack [20]; (**d**) Sauvola [21]; (**e**) Su [23]; (**f**) Jia [24]; (**g**) U2-Net [27]; and (**h**) EBTM.

**Figure 9:**
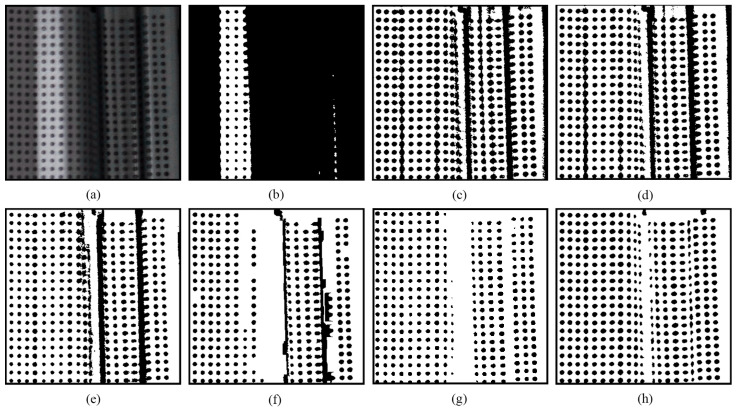
Thresholding of a circular dot image with a wrinkled surface: (**a**) original; (**b**) Otsu [13]; (**c**) Niblack [20]; (**d**) Sauvola [21]; (**e**) Su [23]; (**f**) Jia [24]; (**g**) U2-Net [27]; and (**h**) EBTM.

Figure 8 presents another example where some circular dots were damaged by friction introduced during the stamping process (the arrow points to the friction damage in Figure 8a). It can be seen that the novel approach produces the best thresholding results, as shown in Figure 8h. In Figure 9, the sheet metal surface is wrinkled, making it difficult to correctly separate the circular dots from the background, especially those located in the groove areas (Figure 9b–g). However, the novel approach successfully separates almost all of the visible circular dots, as shown in Figure 9h. These examples highlight the good performance of the novel approach.

Table 1 presents the quantitative statistics of the segmented circular dots, with an average fitting error $e_{mean}\leq 1.0$ and a maximum fitting error $e_{\max}\leq 3.0$. It can be observed that the novel approach achieves the highest quantity of segmented circular dots across all tested images. Specifically, for the circular dot image shown in Figure 6a, which is degraded by low contrast, strong noise, and shadows, the method of U2-Net [27] yields better thresholding results, correctly separating 376 circular dots. In comparison, the novel approach successfully separates 561 circular dots, marking an increase of 49.2%. For the circular dot image in Figure 9a, which is subject to similar challenges, the method of U2-Net [27] achieves better thresholding results with 305 correctly separated circular dots. The novel approach, however, successfully separates 414 circular dots, resulting in an increase of 35.73%. According to Table 2, we can draw the conclusion that the novel approach enhances the quantity of segmented circular dots by over 30% when the circular dot image is affected by low contrast, strong noise, and shadows.

**Figure 10:**
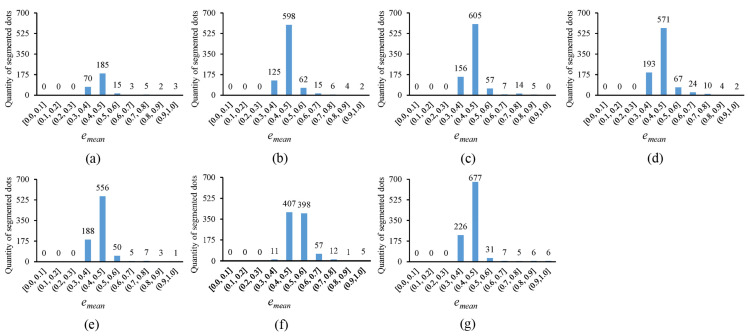
Error histograms of the thresholding outputs presented in Figure 4. (**a**–**g**) show the error histograms corresponding to the thresholding outputs in Figure 4a–g.

**Figure 11:**
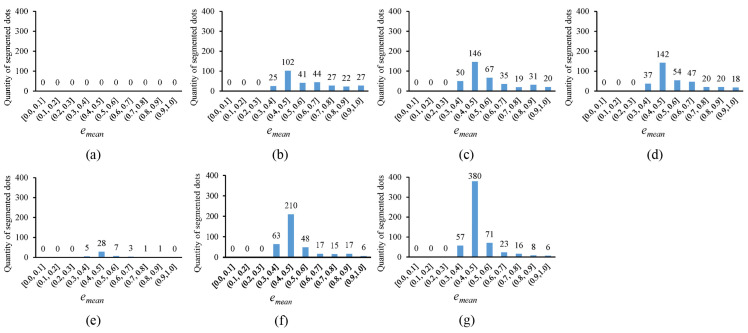
Error histograms of the thresholding outputs presented in Figure 6. (**a**–**g**) show the error histograms corresponding to the thresholding outputs in Figure 6b–h.

**Figure 12:**
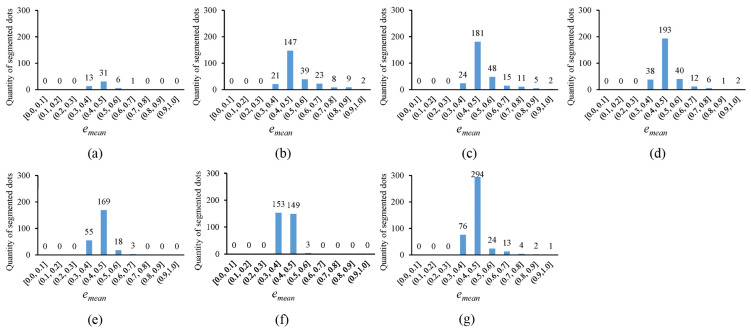
Error histograms of the thresholding outputs presented in Figure 9. (**a**–**g**) show the error histograms corresponding to the thresholding outputs in Figure 9b–h.

In Figure 10, the error histogram, with the horizontal axis representing the average fitting error $e_{mean}$ and the vertical axis showing the quantity of segmented circular dots whose average fitting errors fall within corresponding error ranges, is plotted for the thresholding outputs in Figure 4. Notably, the more segmented circular dots that fall into the smaller error ranges, the better the thresholding method. It can be observed that the novel approach outperforms existing methods, as the segmented circular dots primarily fall into the smaller error ranges of (0.3, 0.4] and (0.4, 0.5]. In Figure 11, the quantity of segmented circular dots (obtained by the novel approach) falling within the smaller error ranges of (0.3, 0.4] and (0.4, 0.5] increases by more than 60%. Furthermore, in Figure 12, the quantity of segmented dots (also obtained by the novel approach) falling into the smaller error ranges of (0.3, 0.4] and (0.4, 0.5] is much more than the other methods. These examples demonstrate the superiority of the novel approach (note that there are no circular dots falling into the error ranges of [0.0, 0.1], (0.1, 0.2], and (0.2, 0.3]; the reason is that only pixel edges are extracted to fit ellipses).

Table 2 presents the thresholding time statistics for the tested circular dot images. The measurements were taken on a laptop equipped with 8 Intel Core i7-1051U CPUs and 16GB of memory. To ensure fairness, all methods were executed on a single core. Since the executable program developed by the authors did not include a time-tracking feature [41], the thresholding time for Jia [24] was not recorded. Additionally, the source code of U2-Net [27], provided by the authors, was implemented in python [42]; for consistency, the thresholding time for U2-Net [27] was also not collected. It can be observed that the method of Otsu [13] is extremely fast, requiring only a few milliseconds to process the tested circular dot images. The novel approach is slower than the locally adaptive thresholding methods of Niblack [20] and Sauvola [21], but it is much faster than the method of Su [23]. When the parallel computing technique OpenMP was employed, the novel approach demonstrated notably improved computational efficiency, requiring only 106 milliseconds to process Figure 1a and 65 milliseconds to process Figure 5a.

## 4. Conclusions

This paper presents an enumeration-based thresholding method (EBTM) for degraded circular dot segmentation. The principles of the novel approach are detailed, and the implementation details are outlined. Unlike traditional methods, the novel approach does not focus on selecting optimal threshold values; instead, it selects the best segments from a series of candidate outputs to generate the desired thresholding result. Owing to the enumeration thresholding mechanism, the novel approach is more robust to nonuniform illumination, shadows, low contrast, specular highlights, strong noise, and certain friction damages. Comprehensive comparative studies using real circular dot images demonstrate the superiority of the novel approach.

The limitations of the proposed method can be analyzed from several aspects. First, it is not well suited for scenarios involving circular dots with significant size variations, as it depends on user-specified diameters for these dots. Nevertheless, in most practical applications—such as stereo vision and motion capture—circular targets (dots) are typically designed to maintain a consistent size, allowing the method to remain effective. Second, a smaller value of *h_j_* can introduce background noise, requiring additional filtering to mitigate its effects. Finally, the criterion developed for evaluating the segments in candidate outputs is specifically tailored to circular dot images and is not applicable to other types of images, such as document images, which limits the scope of the novel approach. However, with the development of new criteria, its applications could be expanded, such as blob detection in biomedical imaging. In the future, we will continue to explore the potential applications of the novel approach.

## Figures and Tables

**Table 1:** Quantitative statistics of segmented circular dots.

Images	Quantity of Segmented Circular Dots Using Different Thresholding Methods						
	Otsu [13]	Niblack [20]	Sauvola [21]	Su [23]	Jia [24]	U2-Net [27]	EBTM
Figure 1a	283	812	844	871	810	891	958
Figure 5a	782	1102	1108	1104	1102	993	1142
Figure 6a	0	288	368	338	45	376	561
Figure 7a	1402	2191	2193	2134	2208	239	2563
Figure 8a	721	784	809	786	690	610	1021
Figure 9a	51	249	286	292	245	305	414

**Table 2:** Statistics of thresholding time.

Images	Thresholding Time for Different Methods (ms)				
	Otsu [13]	Niblack [20]	Sauvola [21]	Su [23]	EBTM
Figure 1a	7	71	96	3820	268
Figure 5a	3	23	52	1359	134
Figure 6a	2	12	11	1034	72
Figure 7a	4	56	75	1863	118
Figure 8a	2	28	13	914	69
Figure 9a	1	9	18	511	41

## Data Availability

The data presented in this study are available on request from the corresponding author.
